# Comparison of non-radiographic axial spondyloarthritis and ankylosing spondylitis patients – baseline characteristics, treatment adherence, and development of clinical variables during three years of anti-TNF therapy in clinical practice

**DOI:** 10.1186/s13075-015-0897-6

**Published:** 2015-12-24

**Authors:** Johan K. Wallman, Meliha C. Kapetanovic, Ingemar F. Petersson, Pierre Geborek, Lars Erik Kristensen

**Affiliations:** Section of Rheumatology, Department of Clinical Sciences Lund, Lund University, Lund, Sweden; Section of Orthopedics, Department of Clinical Sciences Lund, Lund University, Lund, Sweden; The Parker Institute, Department of Rheumatology, Copenhagen University Hospital, Frederiksberg and Bispebjerg, Denmark; Reumatologiska kliniken, Skånes Universitetssjukhus Lund, Kioskgatan 3, 22185 Lund, Sweden

**Keywords:** Spondyloarthritis, Axial spondyloarthritis, Non-radiographic axial spondyloarthritis, Ankylosing spondylitis, Anti-TNF, Treatment outcome, Medication adherence, C-reactive protein

## Abstract

**Background:**

The relationship between non-radiographic axial spondyloarthritis (nr-axSpA) and ankylosing spondylitis (AS) is currently debated. Using observational data from the South Swedish Arthritis Treatment Group register, we thus aimed to compare clinical development and treatment adherence between nr-axSpA and AS patients during three years of anti-TNF (tumor necrosis factor) therapy in clinical practice, and to explore the impact of inflammatory activity measured by CRP (C-reactive protein) at treatment initiation.

**Methods:**

Nr-axSpA and AS patients (n = 86/238) in southern Sweden, commencing anti-TNF therapy 1999-2011, were followed during three years. Anti-TNF cessation was defined as stopping therapy, without restarting another anti-TNF agent within three months. Differences in the three year developments of patient’s visual analogue scale (VAS) scores for global health and pain, EuroQol 5-Dimensions utility, evaluator’s global disease activity assessment, CRP, and ESR (erythrocyte sedimentation rate) were assessed by repeated ANOVA. Anti-TNF adherence was compared by Log rank test and Cox regression. In a subanalysis, the same outcomes were studied after splitting both groups into patients with/without baseline CRP elevation.

**Results:**

Nr-axSpA patients were more often female and had lower acute phase reactants at baseline. Apart from CRP, which remained lower in the nr-axSpA group throughout follow-up (p = 0.004), no between-group differences were detected regarding clinical developments (p >0.1 for all comparisons) or anti-TNF adherence (hazard ratio: 1.1 (95 % CI 0.7 to 1.8) for the nr-axSpA vs. AS group) during three years. Elevated baseline CRP was similarly associated with superior clinical outcomes and treatment adherence in both groups.

**Conclusions:**

With the exception of constantly lower CRP levels in the nr-axSpA group, three years anti-TNF therapy resulted in similar clinical outcomes and treatment adherence in nr-axSpA and AS patients, thus strengthening the hypothesis that these diagnoses represent different aspects/phases of the same disease.

**Electronic supplementary material:**

The online version of this article (doi:10.1186/s13075-015-0897-6) contains supplementary material, which is available to authorized users.

## Background

Spondyloarthritis (SpA) comprises a range of rheumatic disorders - ankylosing spondylitis (AS), undifferentiated SpA, psoriatic arthritis, arthritis related to inflammatory bowel disease, reactive arthritis, and a juvenile form - sharing common clinical features, extraarticular manifestations, and a genetic association with the type 1 major histocompatibility complex HLA-B27. Typical musculoskeletal symptoms are divided into peripheral (arthritis, enthesitis, dactylitis) or axial (sacroiliitis, spondylitis) disease, of which patients may suffer from either or both. AS, the classical form of axial SpA, is defined by structural damage of the sacroiliac joints on conventional radiographs [1]. Many patients, however, display similar axial symptoms and signs of active sacroiliitis on magnetic resonance imaging (MRI) in the absence of such radiographic changes [2], entailing a risk of delayed or missed diagnosis. Thus, in 2009, the Assessment of SpondyloArthritis International Society (ASAS) developed novel classification criteria for axial SpA, encompassing patients both with (AS) and without radiographic sacroiliitis - the latter phenotype thereby formally characterized as non-radiographic axial SpA (nr-axSpA) [3].

Since then a number of studies have compared subjects with nr-axSpA and AS, finding similar levels of disease activity, overall physical impairment, and health-related quality of life (HRQoL), while the nr-axSpA patients are more often female, have shorter mean disease duration, and display lower levels of acute phase reactants and less spinal immobility [2, 4–10]. The observation that 10–12 % of nr-axSpA patients progress to develop AS within 2 years, strengthens the hypothesis that the two entities represent different aspects/phases of a common pathology [9, 11].

Current treatment guidelines for axial manifestations of SpA, including both AS and nr-axSpA, recommend the use of anti-TNF (tumor necrosis factor) agents in cases of insufficient response or intolerance to non-steroidal anti-inflammatory drugs (NSAIDs), as conventional disease-modifying anti-rheumatic drugs (DMARDs) have not been shown to be effective in this situation [12–14]. The efficacy of anti-TNF treatment in nr-axSpA has been shown to be similar to that in AS, at least in patients with objective signs of inflammation, such as elevation of C-reactive protein (CRP) or inflammatory lesions seen on MRI at baseline [5, 9, 15, 16].

While acknowledging these important previous findings, the overall evidence base concerned with nr-axSpA remains fairly limited, and results should be confirmed in further cohorts. Data on long-term efficacy and adherence to anti-TNF therapy in nr-axSpA is also largely absent. In light of this, we thus applied observational data from southern Sweden, aiming to compare baseline characteristics, anti-TNF adherence, and development of clinical variables during three years of anti-TNF therapy between nr-axSpA and AS patients treated in clinical practice.

## Methods

### Patients

Since the late 1990s, patients with chronic arthritis treated with biologic DMARDs in southern Sweden have been monitored in the observational SSATG (South Swedish Arthritis Treatment Group) register, involving 12 rheumatology centers [17]. For the present study, bionaive patients (≥15 years) with nr-axSpA (n = 86) or AS (n = 238), commencing anti-TNF treatment between 1999 and 2011, were retrieved from the SSATG register and compared for baseline characteristics and clinical development during 3 years of anti-TNF therapy. AS patients had a clinical diagnosis according to treating rheumatologists, which in a previous SSATG validation study corresponded to at least 90 % fulfillment of the modified New York criteria (the remaining patients had either incomplete or missing information in their medical records, unabling a validation of the AS diagnosis) [1, 18]. The included nr-axSpA patients were all followed at the central SSATG university clinic in Lund/Malmö, did not have a clinical diagnosis of psoriatic arthritis, and - based on data collected on initiation of anti-TNF therapy - fulfilled the ASAS classification criteria for axial spondyloarthritis, without having skin psoriasis or radiographic sacroiliitis on conventional radiographs (48 and 38 patients, respectively, according to the imaging and clinical arms) [3]. In Sweden, anti-TNF agents were often used to treat patients with this undifferentiated, axial SpA phenotype even before it was formally characterized by ASAS in 2009, explaining the earlier anti-TNF therapy start dates of some patients (31 of 86 nr-axSpA patients commencing anti-TNF therapy prior to 2009).

### Treatment

Anti-TNF therapy was started due to high disease activity and inadequate response or intolerance to one or more NSAIDs. Treatment decisions were taken by the responsible rheumatologists, and no formal disease activity level was required for initiation of anti-TNF therapy. Indications in both nr-axSpA and AS were, however, supported by guidelines when they began to emerge [12, 19], with the non-mandatory Swedish guidelines recommending a Bath ankylosing spondylitis disease activity index (BASDAI) score ≥4 during ≥4 weeks and previous treatment attempts with ≥2 NSAIDs during >3 months in total before starting anti-TNF therapy in both conditions [14]. Administration intervals and dosing of etanercept, adalimumab, golimumab, and certolizumab pegol were in general as recommended by the manufacturers. Infliximab was started at 3 mg/kg at weeks 0, 2, 6, and then every 8 weeks, although in cases of insufficient response, dose increments were allowed in steps of 100 mg to a maximum of 500 mg every 4–8 weeks. Facing adverse events, inefficacy, or other causes, decisions to discontinue anti-TNF therapy altogether or switch to another agent were left to the responsible rheumatologists according to clinical practice.

Patients could receive NSAIDs, low dose oral glucocorticoids, intra-articular glucocorticoid injections, and conventional DMARDs both prior to and during the study period according to clinical practice - the latter mainly applied in patients with peripheral arthritis.

### Follow up and study outcomes

At the start of anti-TNF therapy, demographic data, disease characteristics (allowing for disease classification as outlined above), and information on past and present treatments were reported according to a structured protocol. [17] Patients were followed for 3 years, with scheduled visits at baseline, 3, 6, 12, 24, and 36 months of follow up. At each visit, disease activity was measured by patient’s visual analog scales (VAS) for global health and pain (VAS global and VAS pain), evaluator’s global assessment on a 5-grade Likert scale (Evaluator’s global), and acute phase reactants (erythrocyte sedimentation rate (ESR) and CRP), while HRQoL was estimated as EuroQol 5-Dimensions (EQ-5D) utility, a metric anchored at 1 (full health) and 0 (death), applying the standard British EQ-5D preference set [20, 21]. Use of the BASDAI and Bath ankylosing spondylitis functional index (BASFI) were also recommended [22, 23], although not mandatory within the SSATG during the study period, leading to numbers of missing data values that were too high for meaningful analysis beyond the presentation of baseline values.

Thus, the primary outcomes of the present study were between-group differences (nr-axSpA vs. AS) in the developments of VAS pain, VAS global, Evaluator’s global, ESR, CRP, and EQ-5D during 3 years from the start of anti-TNF treatment. Adherence to anti-TNF therapy was also compared as a secondary outcome. For this analysis, switches between anti-TNF therapies occurring within 3 months were allowed and regarded as continuous treatment, whereas anti-TNF cessation was defined as discontinuing one treatment course without starting another within 3 months.

### Statistics

Baseline characteristics in the nr-axSpA and AS patients were compared by chi square (χ^2^) or Fisher’s exact test for categorical variables and the Mann–Whitney *U* test for continuous variables. Whether the two groups differed in the development of clinical parameters during 3 years of anti-TNF therapy were assessed by repeated analysis of variance (ANOVA), including all study time points and adjusting for sex, age, disease duration, presence of peripheral arthritis (yes/no), and baseline CRP (the latter excluded from analyses of ESR and CRP development). In the main analysis, last observation carried forward (LOCF) was applied to impute missing data, as well as from anti-TNF cessation (without restarting anti-TNF therapy within 3 months) in order to exclude potential effects of later treatments. For EQ-5D, due to a relatively high proportion of missing data, missing values at baseline (n(nr-axSpA/AS) = 40/60) and 3 months (n(nr-axSpA/AS) = 46/81) were first imputed by group-wise linear regression models with sex, age, disease duration, peripheral arthritis status (yes/no), VAS global, VAS pain, evaluator’s global, and health assessment questionnaire (HAQ) scores at the respective time points as covariates [24]. Repeated ANOVA restricted to observed data from patients remaining on anti-TNF treatment was also conducted for sensitivity analysis.

Adherence to anti-TNF therapy was compared by Kaplan-Meier curves and the log rank test, and Cox proportional hazards regression, adjusting for age, sex, disease duration, presence of peripheral arthritis, and baseline CRP, was also applied to derive a between-group hazard ratio.

As a sub-analysis, we then split both patient groups into subjects with (CRP >3.0 mg/l; % (nr-axSpA/AS) = 58/81) or without CRP elevation at baseline, and compared anti-TNF adherence and developments of VAS global, VAS pain, EQ-5D, and evaluatior’s global between the two subgroups within each diagnosis. Statistics were as described above, although limited to analyses of LOCF imputed data and excluding adjustments for baseline CRP. Finally, anti-TNF adherence of all four subgroups were compared by the log rank test.

### Ethics, consent and permissions

Ethical approval for the SSATG register study has been granted by the Regional Ethics Committee at Lund University, and informed consent was given orally by all patients before SSATG enrolment. Due to its quality control character, the SSATG register is part of the legislative documentation demanded in Sweden, and hence no specific ethical approval was required for the present study.

## Results

### Baseline characteristics

Patients with nr-axSpA were significantly younger and had a shorter mean disease duration and fewer previous and ongoing conventional DMARDs than their counterparts with AS (Table 1). The male predominance was also less pronounced in the nr-axSpA group. For disease activity all patient-reported outcomes were similar between the two groups at initiation of anti-TNF therapy, whereas the more objective measures - evaluator’s global, ESR and CRP - were significantly higher among the AS patients.

**Table 1:** Patient characteristics at initiation of anti-TNF therapy

Baseline characteristics	nr-axSpA, n = 86	AS, n = 238	*P* value^a^
Male sex, n (%)	53 (62)	180 (76)	0.013
Age, years	38 (13)	43 (12)	<0.001
Disease duration, years	9 (9)	16 (12)	<0.001
Peripheral arthritis, n (%)	39 (45)	118 (50)	0.501
VAS global, mm	60 (19)	61 (22)	0.313
VAS pain, mm	60 (22)	62 (22)	0.292
Evaluator’s global, score 0–4	1.7 (0.6)	2.0 (0.7)	0.001
BASDAI score	5.3 (1.7)	5.4 (1.9)	0.727
BASFI score	4.1 (2.3)	4.3 (2.1)	0.503
ESR, mm/h	23 (21)	28 (23)	0.026
CRP, mg/l	10 (13)	23 (26)	<0.001
Elevated CRP, n (%)^b^	47 (58)	177 (81)	<0.001
EQ-5D (UK)			0.381
Mean (SD)	0.43 (0.32)	0.45 (0.33)	
Median (IQR; range)	0.59 (0.64; –0.22 to 0.80)	0.62 (1.3; –0.48 to 0.80)	
Concomitant DMARD (%)	29 (34)	150 (63)	<0.001
Previous DMARDs (n)	0.8 (0.9)	1.5 (1.0)	<0.001
First anti-TNF agent used, n (%)			<0.001^c^
Infliximab	19 (22)	112 (47)	
Etanercept	37 (43)	89 (37)	
Adalimumab	20 (23)	37 (16)	
Golimumab	7 (8)	0 (0)	
Certolizumab pegol	3 (4)	0 (0)	

Mean (SD) if not otherwise stated. Missing data in the non-radiographic axial spondyloarthritis (*nr-axSpA*)/ankylosing spondylitis (*AS*) groups, n (%) as follows: Disease duration 1(1)/0(0); visual analog scale (*VAS*) global 7(8)/24(10); VAS pain 7(8)/24(10); Evaluator’s global 2(2)/18(8); Bath ankylosing spondylitis disease activity index (*BASDAI*) 36(42)/105(44); Bath ankylosing spondylitis functional index (*BASFI*) 35(41)/108(45); erythrocyte sedimentation rate (*ESR*) 4(5)/19(8); C-reactive protein (*CRP*) 5(6)/20(8); EuroQ ol 5-Dimensions (*EQ-5D*) 40(47)/60(25); Concomitant disease-modifying anti-rheumatic drug (DMARD) 1(1)/1(0.4). ^a^χ2 for categorical and Mann–Whitney *U* test for continuous variables. ^b^CRP >3.0 mg/l. ^c^Fisher’s exact test

### Development during anti-TNF therapy

**Fig. 1:**
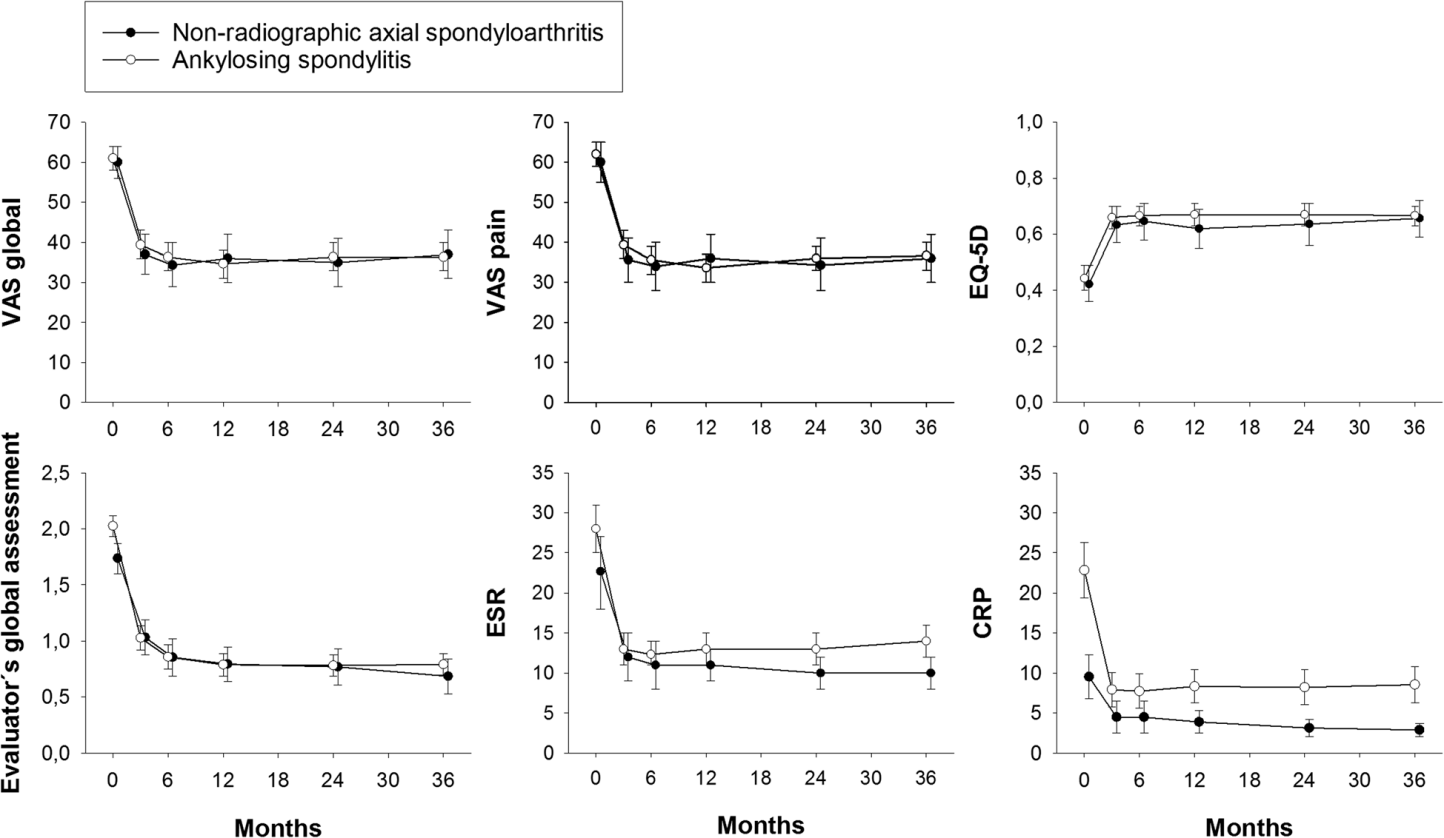
Clinical developments during three years of anti-TNF therapy. Mean (95 % CI) developments in visual analog scale (*VAS*) global, VAS pain, EuroQ ol 5-Dimensions (*EQ-5D*) utility, Evaluator’s global assessment of disease activity, erythrocyte sedimentation rate (*ESR*), and C-reactive protein (*CRP*) in the non-radiographic axial spondyloarthritis and ankylosing spondylitis groups over time, using last observation carried forward imputed data

Following anti-TNF initiation, mean values of VAS global, VAS pain, EQ-5D utility, Evaluator’s global, ESR, and CRP improved rapidly in both nr-axSpA and AS patients, and within 3 to 6 months had reached a plateau, which was then sustained throughout the 3 years of follow up (Fig. 1 and Additional file 1: Figure S1). By repeated ANOVA, regardless of analyzing imputed (Fig. 1) or observed (Additional file 1: Figure S1) data, no between-group differences were seen in the 3-year developments of VAS global, VAS pain, EQ-5D utility, or Evaluator’s global assessment (*p* >0.1 for all comparisons). Point estimate means of ESR and CRP remained higher in the AS patients throughout follow up. For CRP, this between-group difference in the 3-year pattern was statistically significant when analyzing imputed data (*p* = 0.004), while it was only borderline significant in the observed data analysis (*p* = 0.06). ESR development did not differ significantly by either method (*p* >0.1 for both analyses).

### Adherence to anti-TNF therapy

**Fig. 2:**
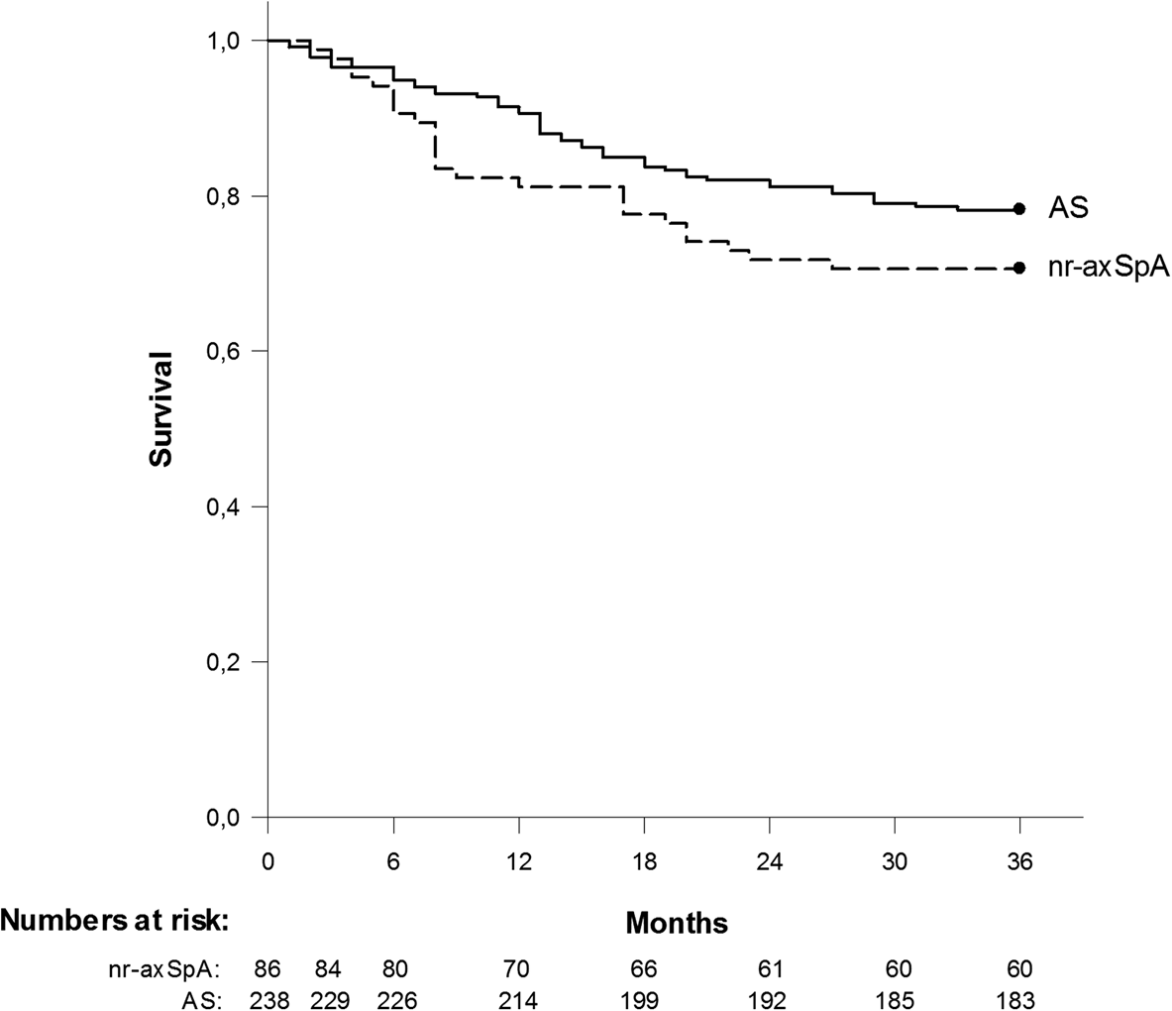
Adherence to anti-TNF therapy. Kaplan-Meier survival curves from initiation of anti-TNF therapy, showing adherence to anti-TNF treatment in the non-radiographic axial spondyloarthritis (*nr-axSpA*) and ankylosing spondylitis (*AS*) groups, respectively. Cessation of anti-TNF therapy was defined as stopping therapy without restarting another anti-TNF agent within 3 months. Numbers of patients still on anti-TNF treatment at the respective time points are shown below the graph

After three years of follow up, the proportions of patients with nr-axSpA (n = 60; 70 %) and AS (n = 183; 77 %) remaining on anti-TNF therapy did not differ significantly (Fig. 2; log rank test, *p* = 0.2, Cox proportional hazard ratio 1.1 (95 % CI 0.7 to 1.8) for the nr-axSpA as compared to the AS group). Male sex and higher baseline CRP levels were significantly associated with better treatment adherence.

Of the 60 nr-axSpA patients adhering to therapy at 3 years, 46 were still receiving their first anti-TNF agent, 11 had switched treatment once, and 3 had switched treatment twice within 3 months. The corresponding figures in the AS group were 155, 24, and 4 patients, respectively. Of the 26 nr-axSpA and 55 AS patients ceasing anti-TNF therapy prior to 3 years, 6 and 7 patients, respectively, did so after having switched treatment once within 3 months, and 2 additional AS patients quit treatment after having switched anti-TNF agents twice.

When only focusing on the first anti-TNF agent used, the adherence rate over 3 years was better in the AS group when analyzed by the log rank test (*p* = 0.04). By Cox regression analysis, however, no significant between-group difference was observed (hazard ratio 1.3 (95 % CI 0.9 to 1.9), whereas male sex and higher baseline CRP again significantly predicted superior treatment retention.

### Baseline CRP sub-analysis

**Fig. 3:**
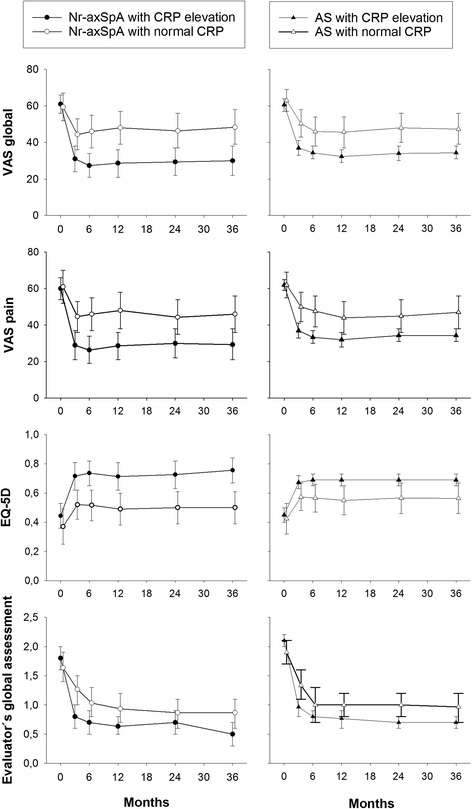
Clinical developments according to C-reactive protein (*CRP*) status at baseline. Mean (95 % CI) developments in visual analog scale (*VAS*) global, VAS pain, EuroQ ol 5-Dimensions (*EQ-5D*) utility, and Evaluator’s global assessment of disease activity during 3 years of anti-TNF therapy in patients with non-radiographic axial spondyloarthritis (*nr-axSpA*) and ankylosing spondylitis (*AS*), who had CRP elevation (CRP >3.0 mg/l) or did not have CRP elevation (CRP ≤3.0 mg/l) at baseline

Among both nr-axSpA and AS patients, significantly larger improvements were seen on repeated ANOVA in VAS global, VAS pain, and EQ-5D utility scores among subjects with elevated CRP at baseline (*p* <0.05 for all comparisons; Fig. 3). For Evaluator’s global assessment, this was also true within the nr-axSpA group, while it was only borderline significant among AS patients (*p* = 0.06).

**Fig. 4:**
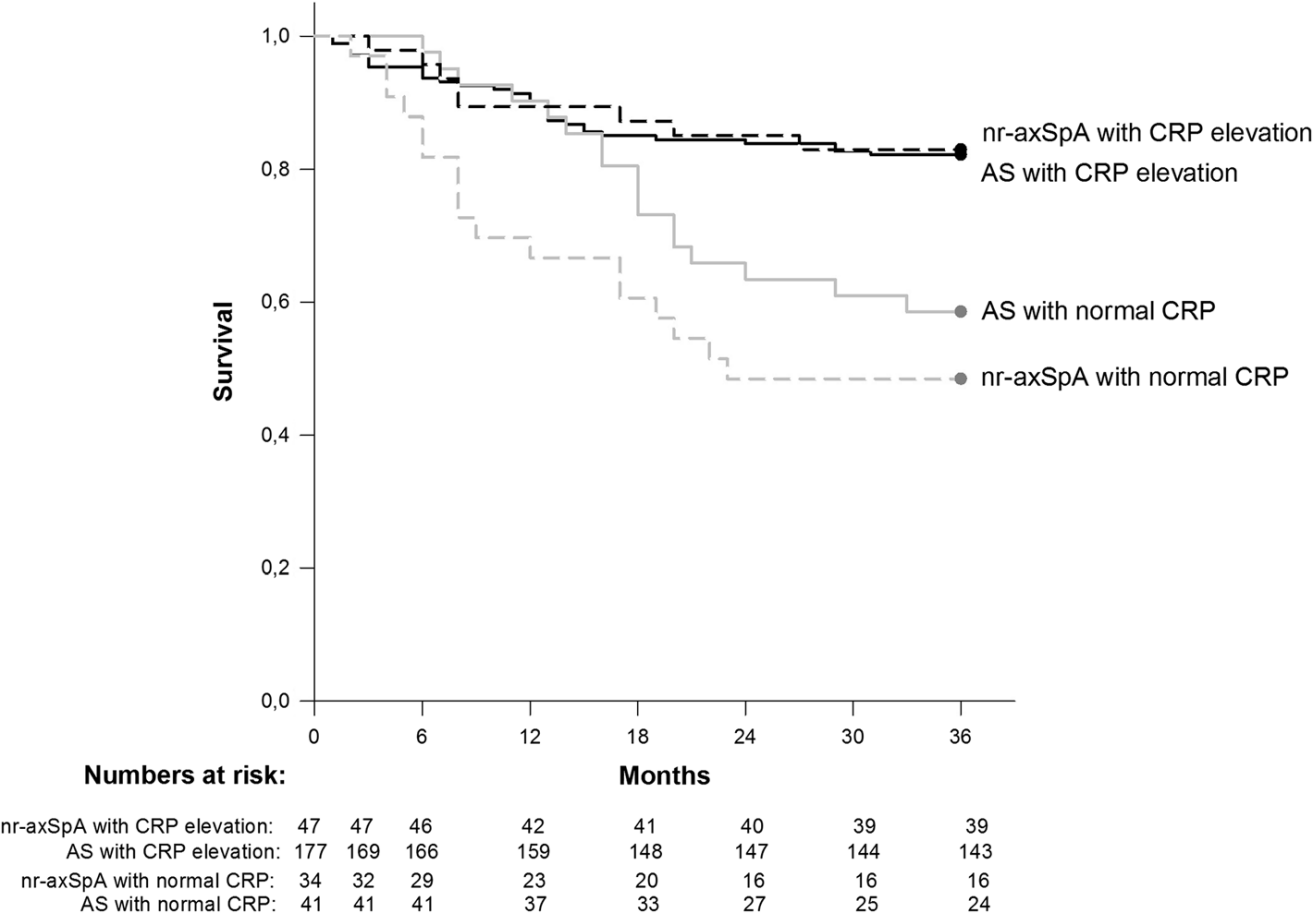
Adherence to anti-TNF therapy according to C-reactive protein (*CRP*) status at baseline. Kaplan-Meier survival curves from initiation of anti-TNF therapy, showing adherence to anti-TNF treatment in patients with non-radiographic axial spondyloarthritis (*nr-axSpA*) and ankylosing spondylitis (*AS*) who had CRP elevation (CRP >3.0 mg/l) or did not have CRP elevation (CRP ≤3.0 mg/l) at baseline. Cessation of anti-TNF therapy was defined as stopping therapy, without restarting another anti-TNF agent within 3 months. Numbers of patients still on anti-TNF treatment at the respective time points are shown below the graph

Likewise, anti-TNF adherence during 3 years of follow up was also significantly superior among both nr-axSpA and AS patients with increased CRP at baseline (Fig. 4; nr-axSpA: log rank test, *p* <0.001; Cox proportional hazard ratio 0.2 (95 % CI 0.1 to 0.6) for patients with CRP elevation, as compared to those without; AS: log rank test, *p* = 0.006, Cox proportional hazard ratio 0.5 (0.3 to 0.9)). When comparing all four subgroups, drug survival did not differ significantly between nr-axSpA and AS patients either with, or without CRP elevation at baseline, whereas the two CRP-positive subgroups both outperformed the CRP-negative groups.

## Discussion

### Main findings

In this study, comparing outcomes of anti-TNF therapy in patients with nr-axSpA and AS treated in clinical practice, despite baseline characteristics indicating more longstanding and objectively more severe disease in the AS group, no between-group differences were observed in adherence to anti-TNF therapy, or in developments of VAS global, VAS pain, EQ-5D utility, or Evaluator’s global assessment of disease activity over 3 years. Mean ESR and CRP levels remained numerically higher in the AS group throughout follow up, although only significantly so for CRP.

Moreover, when splitting patients according to CRP status at baseline (normal vs. elevated), similar outcome patterns were again observed in the two groups, with significantly better treatment adherence and improvements in clinical variables among both nr-axSpA and AS patients with CRP elevation. The diversion of anti-TNF adherence curves observed between the overall nr-axSpA and AS groups (Fig. 2), although non-significant, were thus explained by the relatively large proportion of nr-axSpA patients displaying normal CRP at treatment initiation.

### Previous research

The present comparison of baseline characteristics mainly confirm previous findings, with more female patients and lower levels of acute phase reactants in the nr-axSpA group, whereas no differences were seen in patient-reported disease activity, function, or HRQoL [2, 4–8, 25]. The observed differences in age (absent from most prior comparisons) and disease duration (present in most prior comparisons) should be interpreted with caution, because this was not an inception cohort. The larger proportions of patients with baseline CRP elevation in our study than in most former studies may be due to varying cutoff levels and inclusion criteria, but the intergroup relation remained similar to those previously reported [2, 4–6, 25]. Although the relationship between nr-axSpA and AS remains fiercely debated, there is some evidence that male sex and CRP elevation may predict the development of radiographic changes [9]. According to this hypothesis, in particular women with low levels of inflammation may thus remain in the non-radiographic stage for many years (perhaps lifelong), thereby potentially explaining both the baseline differences seen at the group level and the relatively long mean disease duration observed in the nr-axSpA cohorts of this (9 years) and other studies.

The mean baseline BASDAI, BASFI, and VAS global scores in our cohort are all in the lower ranges of those reported in randomized controlled trials (RCTs) of anti-TNF therapy in nr-axSpA and AS [26–32]. Reasons for this are likely to reside in the observational setting of our study, without strict inclusion criteria or any mandatory requirement based on disease activity, to start anti-TNF therapy. The between-group differences in the first anti-TNF agent used (Table 1) reflect the earlier approval of this treatment indication in AS. Although no head-to-head studies have been performed, there is no evidence to support a difference in efficacy of the various TNF inhibitors on manifestations of axial or peripheral SpA [13, 33].

Direct comparisons of the effects of anti-TNF therapy in patients with nr-axSpA and AS have hitherto been published from two RCTs and one observational cohort [5, 15, 16, 25, 34]. In line with the current results, these studies have demonstrated similar treatment response rates in both groups, at least in the presence of comparable signs of inflammation at baseline, such as elevation of CRP or inflammatory lesions seen on MRI - both identified as predictors of anti-TNF response in patients with nr-axSpA [30–32, 35], and in AS [36–41]. A recent meta-analysis, assessing all available RCT evidence, also came to a similar conclusion, with comparable greater anti-TNF therapy effect sizes over placebo in both diagnoses when adjusting for publication year as a proxy of disease severity (as early AS trials enrolled patients with more longstanding disease) [26]. In terms of effects on HRQoL, developments in the EQ-5D during anti-TNF treatment of patients with nr-axSpA and AS have not been previously compared, although the available 2-year Short Form 36 and AS quality of life data also point to comparable HRQoL gains in both groups [16]. Contrasting with the present results, in the RCT by Song et al., no difference was observed between the nr-axSpA and AS groups in development of CRP [34]. Inclusion in that trial, however, required the presence of inflammatory lesions seen on MRI, and as opposed to most other comparisons [2, 5, 7, 25], mean CRP levels also did not differ at baseline.

Apart from a previous report from our group, finding inflammatory sacroiliitis lesions on MRI to predict better drug survival of a first TNF inhibitor in nr-axSpA [42], data on anti-TNF adherence in this diagnosis remain sparse. In contrast to our findings, based on retrospective data, Wallis et al. reported indications of worse adherence to anti-TNF therapy among patients with nr-axSpA as compared to AS [7], but prospectively conducted comparisons are still lacking. In line with the present results, however, male sex and CRP elevation at baseline have been shown to predict better adherence to anti-TNF therapy in patients with AS [43, 44].

In view of the relatively worse outcomes among both patient groups without CRP elevation, it may be hypothezised that in some of these subjects comorbidity in the form of chronic widespread pain (CWP) was falsely interpreted as spondyloarthritis activity, thus contributing to the decision to start anti-TNF therapy but not responding to this treatment. On the contrary, in patients with more active SpA - as reflected, for example, by CRP elevation - a certain improvement with anti-TNF therapy would be expected regardless of the presence of concomitant CWP or not. While the clinical arm of the ASAS axial SpA classification criteria has met with repeated concern that it may falsely classify CWP patients as having spondyloarthritis [45, 46], in a recent study presented in abstract form, no patients with primary fibromyalgia did indeed meet these criteria [47]. On the contrary, however, around 15 % of both AS and nr-axSpA patients also fulfilled criteria for fibromyalgia, and other studies have likewise reported a prevalence of concurrent fibromyalgia in AS at around 10–15 % [48, 49]. Furthermore, patient-reported SpA measures are known to differentiate poorly between symptoms related to SpA or CWP [48], with generally worse outcomes among patients with such comorbidity [49, 50], and poorer adherence to anti-TNF therapy was also recently reported in SpA patients with concurrent fibromyalgia [50].

### Strengths and limitations

The relatively long follow up (3 years) and the opportunity to compare outcomes of anti-TNF therapy in patients with nr-axSpA and AS treated in clinical practice within the same observational setting, comprise major strengths of the present study. To our knowledge, a direct comparison of adherence to anti-TNF therapy in patients with these diagnoses based on prospectively collected data has been performed here for the first time. Furthermore, the available data on EQ-5D developments during anti-TNF therapy in patients with nr-axSpA was previously limited to 1-year results from a single study [30].

In terms of limitations, the open, observational setting entails inherent risks of bias in patient selection, assignment of treatment, and collection of clinical data. Moreover, the relatively high proportion of missing data for some outcomes is another frequent problem in observational studies, and for this reason the results of the main analysis are presented based on both imputed and observed data. On the other hand, the observational SSATG setting also implies that patient inclusion will not be restricted by any predefined level of disease activity, by rigid treatment guidelines or economical aspects, but rather will reflect clinical practice. At the same time, all patients were indeed selected for anti-TNF therapy, and results are thus not necessarily generalizable to the nr-axSpA and AS populations at large. The generalizability in nr-axSpA is also compromised by the exclusion of patients with a clinical diagnosis of psoriatic arthritis. For certainty of the diagnoses in our cohort, high validity for AS diagnoses has previously been demonstrated within the SSATG register, while review of the medical records confirmed that all included nr-axSpA patients fulfilled the ASAS classification criteria for axial disease without fulfilling the modified New York criteria. This said, however, a certain misclassification between the two diagnoses cannot be entirely ruled out [51]. Finally, due to their non-mandatory status within the SSATG during the study period, the lack of SpA-specific outcome measures such as BASDAI and BASFI beyond the baseline values is a main drawback of the present study. Based on the limited data available, however, developments in BASDAI closely resembled those of the presented patient-reported outcomes, whereas this was less apparent for BASFI, although the numbers of observations were small.

## Conclusions

In this comparison of patients with nr-axSpA and AS in southern Sweden receiving anti-TNF therapy during 3 years in clinical practice, many similarities, and only a few differences were observed. Patients with nr-axSpA were more often female and had less elevation of acute phase reactants at baseline, with CRP levels remaining significantly lower than in the AS group throughout follow up. In spite of this, patient-reported measures of disease activity and HRQoL did not differ at baseline and followed nearly identical trajectories in both groups during the 3 years of follow up. Furthermore, no between-group difference was detected in adherence to anti-TNF therapy, and CRP elevation at baseline was similarly associated with superior clinical outcomes and anti-TNF treatment persistence in both groups. Overall, the current results thus seem to strengthen the prevailing hypothesis that nr-axSpA and AS represent different aspects/phases of the same underlying disease.

## Additional file

Additional file 1: Figure S1. Clinical developments during three years of anti-TNF therapy based on observed data. (DOCX 264 kb)

## Abbreviations

ANOVA: analysis of variance
AS: ankylosing spondylitis
ASAS: Assessment of SpondyloArthritis International Society
BASDAI: Bath ankylosing spondylitis disease activity index
BASFI: Bath ankylosing spondylitis functional index
CI: confidence interval
CRP: C-reactive protein
CWP: chronic widespread pain
DMARD: disease-modifying anti-rheumatic drug
EQ-5D: EuroQol 5-Dimensions
ESR: erythrocyte sedimentation rate
HAQ: Health Assessment Questionnaire
HLA: human leukocyte antigen
HRQoL: health-related quality of life
IQR: interquartile range
LOCF: last observation carried forward
MRI: magnetic resonance imaging
nr-axSpA: non-radiographic axial spondyloarthritis
NSAID: non-steroidal anti-inflammatory drug
RCT: randomized controlled trial
SD: standard deviation
SpA: spondyloarthritis
SSATG: South Swedish Arthritis Treatment Group
TNF: tumor necrosis factor
UK: United Kingdom
VAS: visual analog scale
