# Supplementary material for: Comparison of non-radiographic axial spondyloarthritis and ankylosing spondylitis patients – baseline characteristics, treatment adherence, and development of clinical variables during three years of anti-TNF therapy in clinical practice
Source: Arthritis Res Ther. 2015 Dec 24;17:378. doi: 10.1186/s13075-015-0897-6 (PMC4718030; doi:10.1186/s13075-015-0897-6)
Supplement: Additional file 1: Figure S1. — Clinical developments during three years of anti-TNF therapy based on observed data. (DOCX 264 kb) [file 13075_2015_897_MOESM1_ESM.docx]

**Additional file 1**

**___________________________________________________**

**Comparison of nonradiographic axial spondyloarthritis and ankylosing spondylitis patients – baseline characteristics, treatment adherence, and development of clinical variables during three years of anti-TNF therapy in clinical practice**

J.K. Wallman^1^, M.C. Kapetanovic^1^, I.F. Petersson^1,2^, P. Geborek^1^, L.E. Kristensen^1,3^

^1^ Section of Rheumatology, Department of Clinical Sciences Lund, Lund University, Lund, Sweden

^2^ Section of Orthopedics, Department of Clinical Sciences Lund, Lund University, Lund, Sweden

^3^ The Parker Institute, Department of Rheumatology, Copenhagen University Hospital, Frederiksberg and Bispebjerg, Denmark


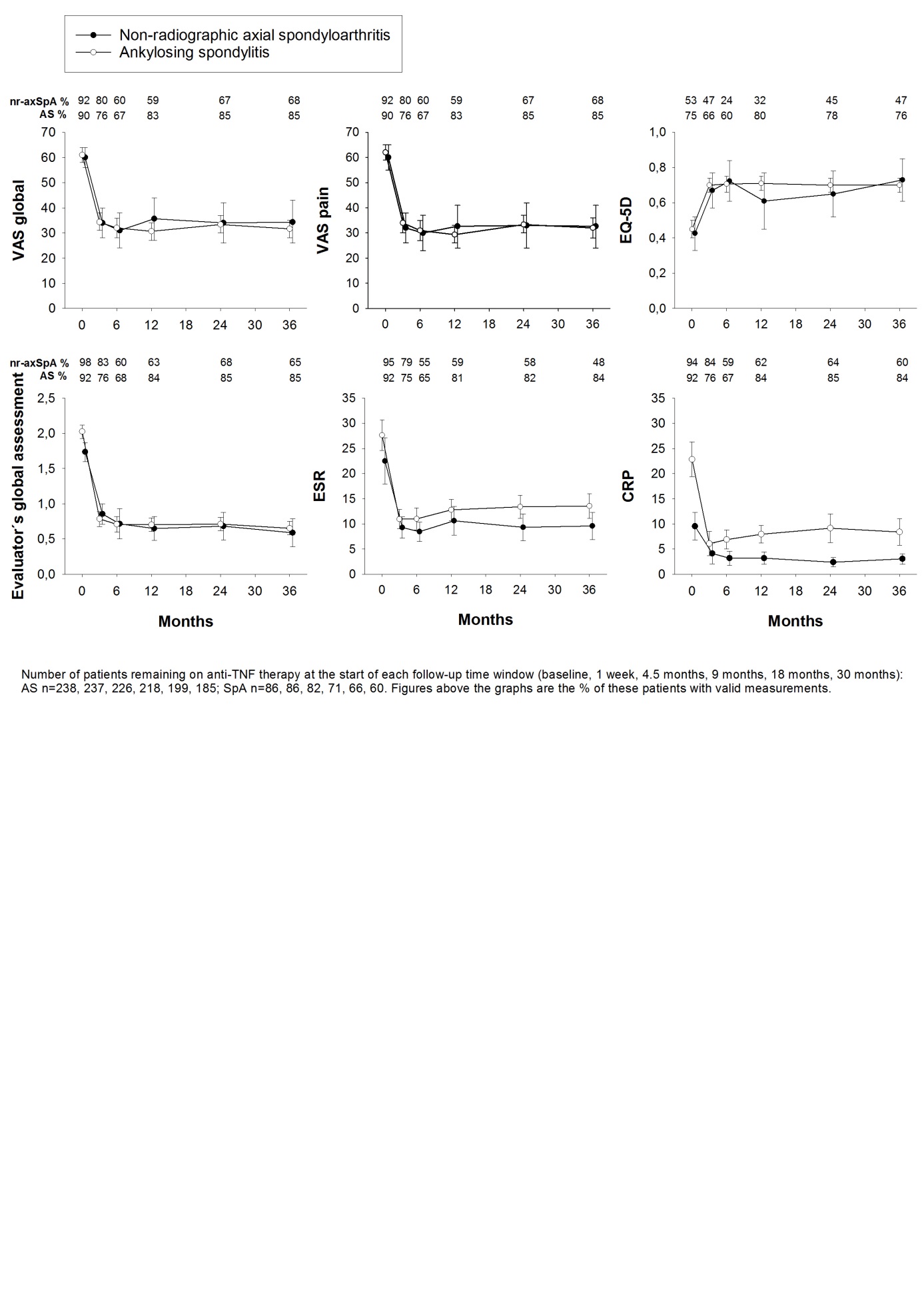


**Supplementary Figure S1.** **Clinical developments during three years of anti-TNF therapy based on observed data.** Mean (95% CI) developments of VAS global, VAS pain, EQ-5D utility, Evaluator´s global assessment of disease activity, ESR, and CRP in the nr-axSpA and AS groups over time, based on observed data from patients remaining on anti-TNF treatment at the start of each follow-up time window (baseline, 1 week, 4.5 months, 9 months, 18 months, 30 months; nr-axSpA n=86, 86, 82, 71, 66, 60; AS n=238, 237, 226, 218, 199, 185). Figures above the graphs are the % of these patients with valid measurements.
